# Prevalence of radiographic detectable intervertebral disc calcifications in Dachshunds surgically treated for disc extrusion

**DOI:** 10.1186/1751-0147-52-24

**Published:** 2010-04-14

**Authors:** Cecilia Rohdin, Janis Jeserevic, Ranno Viitmaa, Sigitas Cizinauskas

**Affiliations:** 1 University Animal Hospital, Department of Clinical Sciences, Swedish University of Agricultural Sciences, Box 7040, 750 07 Uppsala, Sweden; 2 Referral Animal Neurology Hospital Aisti, Virtatie 9, FI-016000, Vantaa, Finland; 3 Estonian University of Life Sciences, Tartu, Estonia

## Abstract

**Background:**

An association between the occurrence of calcified discs, visible on radiographic examination (CDVR), and disc extrusions has been suggested in published literature over the past 10-20 years, mainly from Nordic countries. It has also been postulated that dogs without CDVR would not develop disc extrusions. Furthermore, inheritance of CDVR has been calculated and it has been postulated that, by selecting dogs for breeding with few, or no CDVR, the prevalence of disc extrusions in the Dachshund population may be reduced.

**Methods:**

The prevalence of radiographic detectable intervertebral disc calcifications was calculated from one hundred surgeries for disc extrusion, performed in 95 Dachshunds, in order to determine if the disc causing clinically significant IVDD, had radiographic signs of calcification at the time of confirmed disc extrusion. Inclusion criteria, for each dog, included a complete physical, orthopedic and neurologic examination, radiographs of the entire vertebral column, a myelogram or magnetic resonance imaging examination indicating extradural spinal cord compression, and finally a surgical procedure confirming the diagnosis of a disc extrusion. In addition to descriptive statistics, age correlation with number of calcifications visible at radiographic examination and with CDVR at the surgery site was examined.

**Results:**

We found that disc extrusions occur as frequently in discs that are found to have radiographic evidence of calcification as those discs that do not have signs of radiographic calcification, and that IVDD (intervertebral disc disease) requiring surgery does occur in the absence of any calcified discs on radiographic examination. We found that calcified discs were more frequent in our Dachshund population compared to previous studies suggesting that disc calcification might be a serious risk factor for developing disc extrusion. Further studies are needed to show, conclusively, if selection of breeding dogs based on CDVR in the Dachshund will reduce the incidence of IVDD. The presence of the calcifications of intervertebral disc should be evaluated with caution, as only part of the calcifications will be detected and the real extent of the disc degeneration may be underestimated.

## Background

Intervertebral disc disease (IVDD) in dogs is one of the most common disorders in veterinary neurology. The chondrodystophic (hypochondroplastic) breeds are affected more frequently and in the Dachshunds the breed prevalence of IVDD is around 19% [1-4]. Degenerative changes in the intervertebral discs can be detected already in the newborn hypochondroplastic dog and at the age of one year most of the discs will show chondroid metaplasia [2]. The degeneration can lead to subsequent mineralization (calcification) of the discs. Calcifications have been reported to be present in 46-48% of the intervertebral discs on histological examination in Dachshunds [2,5]. Some of the calcifications will become extensive enough to be visible on plain radiographs, however, only part of the calcifications present on histopathology will be visualized on radiographic examination [5]. Disappearance of radiographic visible disc calcifications (CDVR), due to phagocytic activity, disc extrusion or other process, is known to occur with time [6,7] therefore the occurrence and frequency of CDVR is a dynamic process.

Some authors claim an association between the occurrence of CDVR and disc extrusions [7-14]. It has also been postulated that dogs without CDVR would not develop disc extrusions [8]. Furthermore, inheritance of CDVR has been calculated, supporting a genetic basis for CDVR [9]. However, it has been suggested that a common environmental factor, presumably resulting from non-genetic causes, is significant in determining the number of discs to undergo calcifications in affected dogs [12]. It has been postulated that, by selecting dogs for breeding with few, or no CDVR, the prevalence of disc extrusions in the Dachshund population may be reduced [8,9]. As a result of the published research over the past 10-20 years, mainly from Nordic countries, selective breeding programs have been initiated in Norway, Denmark and Finland. According to the program, Dachshunds with 0-2 CDVRs are accepted for breeding, Dachshunds with 3-4 CDVRs can be accepted for breeding but only to a dog with 0-1 calcifications and finally Dachshunds with more than 5 CDVRs should not be accepted for breeding (Norske Dachshundklubbers Forbund prøveprosjekt 01.05.2002, Dansk Gravhundeklub avlsanbefaling pr. 1.12.2008).

To the author's knowledge, evidence that dogs with higher number of CDVR are predisposed to disc extrusion, is still missing. It is also not proven that only the CDVRs cause disc extrusion and that discs without CDVR do not cause disc extrusion. Therefore it is still questionable if selection of Dachshunds with less CDVR will reduce the incidence of the IVDD in the Dachshund population in the future.

We postulate that intervertebral discs with or without CDVRs can be affected by disc extrusions as the majority of the intervertebral discs are degenerated and calcified, as proven by histopathological studies [2,5]. The aim of the current study is to determine if the disc causing the clinically significant IVDD, had radiographic signs of calcification at the time of confirmed disc extrusion.

## Methods

Dachshunds treated surgically for intervertebal disc extrusion at the Referral Animal Neurology Hospital, Aisti, Helsinki, from 2005-2008, were included in the study. Inclusion criteria, for each dog, included a complete physical, orthopedic and neurologic examination, radiographs of the entire vertebral column, a myelogram or magnetic resonance imaging examination indicating extradural spinal cord compression, and finally a surgical procedure confirming the diagnosis of a disc extrusion.

A complete neurological examination, including neuroanatomic localization, was performed in each dog according to Lorenz and Kornegay [15]. Neurological assessments were made by a diplomate ECVN (SC) or a resident in neurology (CR, JJ and RV). For neurological assessment a modified Frankel spinal cord injury scale was used. Dogs were classified as having spinal hyperesthesia (grade I), ambulatory paraparesis or tetraparesis and ataxia (grade II), nonambulatory paraparesis or tetraparesis (grade III), paraplegia or tetraplegia with present deep pain nociception (grade IV) or paraplegia or tetraplegia with absent deep pain nociception (grade V). A conscious response to severe noxious stimuli by applying a forceps across the digits of the pelvic or thoracic limbs confirmed the presence of deep pain nociception.

Dogs were sedated with medetomidine (Domitor^® ^1 mg/ml) 10-20 μg/kg intramuscularly (IM) and methadone (L-Polamivet^®^) 0.1-0.2 mg/kg IM for the radiographic examination. If sedation was inadequate for perpendicular positioning of the vertebral column to the film, anesthesia was induced with propofol (Propofol^®^) and maintained on isofluran (Isofluran^®^) and O_2_. Lateral and ventrodorsal survey radiographs were taken of the entire vertebral column, from C1 to S3. Eight exposures, 4 lateral and 4 ventrodorsal, using 15 × 30 cm flexible phosphor screen (Kodak, GP) were taken of each column and digitally processed with Kodak direct view CR500. When sedation was adequate for optimal radiographic imaging, anesthesia was not induced before performing the MRI or the myelogram.

Two of the authors (JJ and RV) evaluated the radiographs on E-film (Merge^®^, Healthcare) together and the decisions if the discs were deemed calcified or not, were made in consensus. The images were magnified and contrast was changed in order to achieve optimal impression of the area of interest.

The number of calcified discs was calculated and their localization in the vertebral column was recorded. All visible intervertebral disc calcifications including calcifications of disc material in the intervertebral disc space, the intervertebral foramen and adjacent vertebral canal were calculated as calcifications.

A myelogram was performed by subarachnoid injection of contrast media, Iohexol (Omnipaque^® ^240 mgl/ml) at a dose of 0.3 ml/kg. A lumbar puncture (L5-L6) was performed when the neurologic examination indicated a thoracic or lumbar localization and a cisternal puncture was performed when the neurologic examination indicated a C1-T2 localization. Radiographs were obtained immediately after injection in lateral, ventrodorsal, and oblique views (45°). Myelography interpretation was performed according to Sharp and Wheeler [16].

Magnetic resonance imaging (MRI) was performed with an ESAOTE 0.18 T equipment. Dogs were positioned in left lateral recumbency in a human elbow or shoulder coil. Sagittal and transverse images were acquired using T1W (TR 590-750; TE 18-26; acquisition number 2-3, FOV 192-288/192-224; slice thickness 4-5 mm; gap between slices 0.4-0.5 mm) and T2W (TR 3000; TE 80-90; acquisition number 1-2; FOV 192-288/192-200; slice thickness 4-5 mm; gap between slices 0.4-0.5 mm) sequences. Extruded disc material was characterized by low signal intensity within the epidural space in T1 and T2 weighted images [17]. MRI was the preferred diagnostic modality. However, weather a MRI or a myelogram was performed depended largely on the availability of the MRI equipment, with myelograms commonly carried out outside of normal clinic hours. MRI examinations were also performed in cases when the myelogram was inconclusive.

Following diagnostic imaging, the dogs proceeded directly to surgery. Surgical approach depended on the localization of the affected disc. A ventral decompression (slot) was performed when the disc extrusion was located in the cervical area, or a left or right sided hemilaminectomy was performed when the disc extrusion was located in the thoracic and lumbar area as described by Sharp and Wheeler [18,19]. Disc fenestrations were not performed in any of the surgeries.

In addition to descriptive statistics, age correlation with number of CDVR and with CDVR at the surgery site was examined. For analysis, three age groups were defined: dogs < 5 years of age, 5-7 years of age and 8 years and older. Non-parametric Kruskal-Wallis test followed by Tukey test with ranked sums for pair wise comparisons was used to compare the difference in the number of CDVR between age groups. Differences in proportions of dogs having CDVR at surgery site between age groups pair wise were tested using chi-square test.

The number of CDVR was divided in two groups: 0-4 CDVR and 5 or more CDVR.

Statistical analyses were performed with Stata 10/IC (Statacorp LP, College Station, USA) software. Statistical software WINKS SDA 6.0 (TexaSoft, Cedar Hill, USA) was used for Kruskal-Wallis test. The level of significance was set at 5% (p = 0.05).

## Results

Ninety five dogs were included in the study. Five dogs had two surgeries during the time of the study and their data were evaluated twice as two separate examinations making together 100 patients in the study population. The five Dachshunds, involved in two surgeries, had their second extrusion involving a disc separate from the one previously affected. The median age of affected dogs at the time of examination was 74 months (mean 78.08; SD ± 26.67; range 31, 158 months). The study group included 44 female and 51 male Dachshunds. One female and four male dogs were operated and included twice in the study population. The neuroanatomic lesion localization corresponded to the area of disc extrusion in all cases. The neuroanatomic localization was for 6 dogs at C1-C5, for 88 dogs at T3-L3 and for 6 dogs at the L4-S3 spinal cord segments. Hyperesthesia and paraparesis with decreased to absent postural reactions in the pelvic limbs were the most frequent complaint. The neurological status was assessed, and grade I was found in 3 dogs, grade II in 46 dogs, grade III in 24 dogs, grade IV in 13 dogs and finally grade V in 14 dogs. Subsequently, 97% of the Dachshunds of our study showed neurological deficits related to their disc extrusion.

Two dogs had eight lumbar vertebrae, two **other **dogs had **an **intervertebral disc present between the first and second sacral segments making a total number of 27 intervertebral discs per dog. None of these additional discs were calcified on radiographic examination; therefore they were not included in further calculations. A block vertebrae and consequently an absent intervertebral disc was found in four dogs (between T13-L1 in one and between L6-L7 in three dogs). Therefore, in our study population, a total of 2600 disc spaces were evaluated.

**Figure 1:**
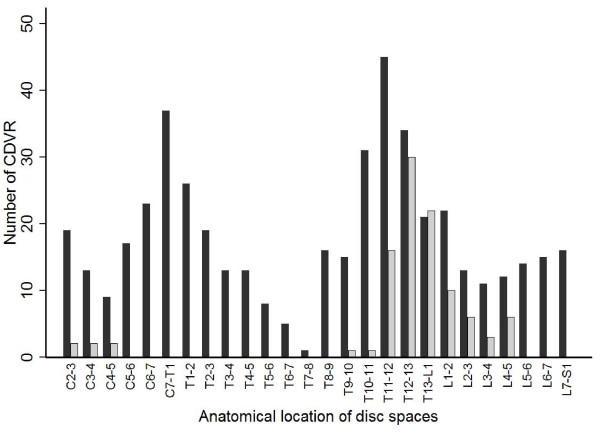
**The number of CDVR (dark grey chart) and surgical sites (pale grey chart) in 100 surgically treated Dachshunds**.

Calcified discs were found in 87 of the 100 radiographic examinations of spines (Figure 1.). In this study population, CDVR were found in all intervertebral disc spaces that contain a disc. Of the 2600 discs examined, 477 (18%) showed signs of calcifications. CDVR were most frequently found at disc-spaces T11-T12 (9.4%), C7-T1 (7.9%), T12-T13 (7.1%), T10-T11 (6.5%) (Figure 1.). The median number of CDVR was 4 (mean 4.77; SD ± 3.77; range 0, 19).

There was no difference in number of calcified discs in female and male Dachshunds. Thirteen dogs had no radiographic visible disc calcifications, 7 dogs had one CDVR, 13 dogs had two CDVR, 10 had three CDVR, 10 had four CDVR and 47 dogs had five or more CDVR. Hence, 53 of the Dachshunds from our study had less than 5 CDVR.

**Figure 2:**
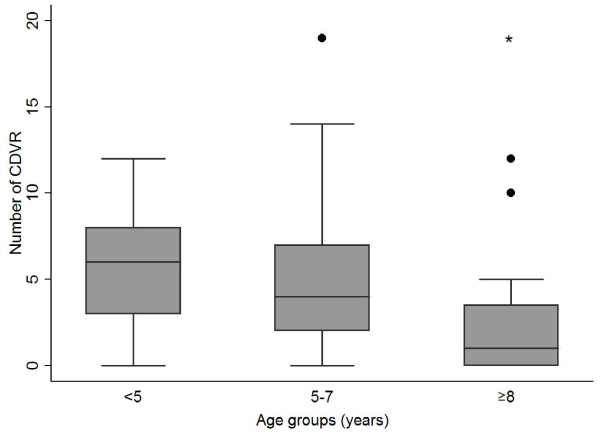
**Box plots presenting number of CDVR in Dachshunds of different age groups**. Black dot, represents outlier dog value within the group. Asterisk, represents significant difference (p < 0.05) compared with younger age groups.

**Figure 3:**
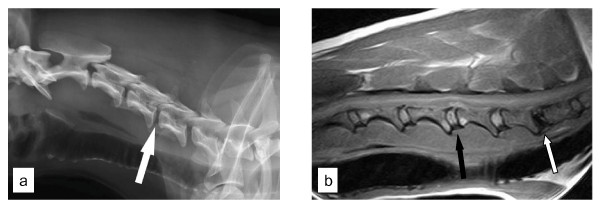
**a. The radiographic examination of the cervical spine in a Dachshund with a surgically confirmed disc extrusion at C4-C5 (white arrow)**. Notice the absence of CDVR at C4-C5. **b. A T1-weighted sagittal magnetic resonance image of the cervical spinal cord of the Dachshund in 3a**. Notice the extent of calcification of the C6-C7 disc (white arrow) compared to the affected disc at C4-C5 (black arrow).

Dogs at an age of 8 years and older, showed significantly less CDVR compared with the younger age groups (Figure 2.). Myelography was performed in 58 cases, magnetic resonance imaging in 35 dogs and in 7 cases both myelography and MRI was needed to identify the site and the side of extrusion. Hansen type I herniation, disc extrusion, was confirmed during all 100 surgeries after performing 6 ventral slots and 94 hemilaminectomies. The disc space's affected with disc extrusion were found to have radiographic calcifications in 54 of the 100 radiographic examinations. Subsequently, in 46% of the Dachshunds the affected disc was not calcified on radiographic examination (Figure 3.). The discs most commonly affected by IVD extrusions were T12-T13 (28%), followed by T13-L1 (23%), T11-T12 (15%) and L1-L2 (10%) (Figure 1.). Dachshunds 8 years and older showed significantly less frequently CDVR at the surgery site compared with the 5 to 7 years old dogs (Table 1).

**Table 1:** Mean number of calcified discs, visible on radiographic examination (CDVR), number of dogs having 4 or less and 5 and more CDVR and dogs having CDVR at surgery site in 100 surgeries for disc extrusion in different age groups.

	≤ 4 years	Age groups 5-7 years	≥ 8 years
Nr of dogs in groups	27	53	20
Mean nr of CDVR ± SD	5.74 ± 3.24^a^	5.17 ± 3.83^a^	2.4 ± 3.42^b^
Nr of dogs with ≤ 4 CDVR	10 (37%)	27 (51%)	16 (80%)
Nr of dogs with ≥ 5 CDVR	17 (63%)	26 (49%)	4 (20%)
CDVR at surgery site	14 (52%)^ab^	35 (66%)^b^	5 (25%)^a^

^a, b ^different superscript letters indicate statistically significant (p < 0.05) difference between age groups within row

Five dogs had two surgeries performed on two separate occasions in the thoracolumbar spine. The mean number of CDVR was 7.2 in these five dogs. The extruded discs had CDVR in 6 of the 10 examinations and in all 5 dogs it was a disc at a different location that extruded and caused repeated signs of paralysis. The number of CDVR was the same in two (8/8, 10/10), decreased in two (3/2, 9/5) and increased in one (8/9) dog in the repeated radiographic examination. The time period between the two surgeries was less than 12 months in 3 dogs (6, 7 and 7 months), 16 months in one and 32 months in another patient. The disc next to the previous operation site was extruded in three cases (Th11-12/Th12-13, L1-2/L2-3, Th13-L1/Th12-13). In the other two cases a more remote disc caused the second extrusion (Th11-12/Th13-L1, Th13-L1/L3-4).

## Discussion

It has been postulated that it is usually intervertebral discs with CDVR that are subjected to Hansen type I herniation [8]. Conversely, our results indicate that disc extrusions occur as commonly in discs that are assessed as calcified on radiographs as in discs without CDVR. In 54% of the Dachshunds in our population, the disc causing the extrusion was calcified on radiographic examination. Interestingly extruded discs were not most often visibly calcified on radiographic examination in the younger age group to which the radiographic evaluation method is adapted, but was most frequent in the middle age group (peak 5 year old dogs). The middle age group, from 5-7 years of age, showed the highest disc extrusion prevalence (53 dogs) in our study population. In 8 years and older dogs extruded discs were less frequently calcified on radiographic examination, following the same trend as the general number of CDVR. It has also been postulated that dogs with no or one radiographic disc calcification might be at a reduced risk of developing disc extrusion [7,9-14] and that disc extrusions do not occur in Dachshunds without CDVR [8]. Our material indicates that IVDD requiring surgery does occur also in Dachshunds without any CDVR. In 13% of the dogs in our study no disc calcifications were found on radiographic examination.

Multiple studies have suggested that screening the vertebral columns of Dachshunds intended for breeding, by radiographic examination, may be valuable in reducing the incidence of disc extrusion [7,9-14]. According to these suggestions, Dachshunds with 0-2 CDVR are accepted for breeding, 3-4 CDVR may be accepted, and dogs with > 5 CDVR should not be used for breeding purposes (Norske Dachshundklubbers Forbund prøveprosjekt 01.05.2002, Dansk Gravhundeklub avlsanbefaling pr. 1.12.2008). In 57% of the Dachshunds treated for disc extrusion in our study, 0-4 calcified discs were found at the time of radiological examination, and would accordingly, have been considered part of a low-risk-population and would have been accepted for breeding purposes according to the suggested scheme. Also the number of CDVR showed statistically significant difference between the three major age groups. The number of CDVR has been reported to be a good predictor of clinically significant IVDD in Dachshunds [9,10,12]. Our case material clearly indicate that Dachshunds without and with rare CDVR will be affected by disc extrusion with the same frequency as Dachshunds with multiple calcifications visible on radiographic examination.

Absence of CDVR on radiographic examination does not mean that the disc is neither degenerated nor calcified. It has been conclusively demonstrated that radiographic examination is considerably less sensitive than histopathology in detection of calcifications within the intervertebral disc calcifications [5]. Histopathology is considered to be the golden standard for detection of disc calcifications. Previous studies have found calcifications on histopathological examination in 46-48% of the intervertebral discs in Dachshunds [2,5]. This would mean that approximately 12 intervertebral discs in the Dachshunds are expected to be calcified according to the histopathological study. In contrary, radiological studies were able to detect 2.5-3.4 [20], 3.5 [21], 3.7 [13], 4.8 (current study) of calcifications in Dachshunds (mean values). This indicates that radiological studies are detecting only about 20-40% of the actually existing disc calcifications that can be identified with histopathology. Computer tomography, a more sensitive diagnostic modality, may detect less calcified discs not visible on conventional radiography and may in this way be of advantage when considering breed screening programs.

The occurrence of intervertebral disc calcifications is not constant throughout the hypochondroplastic dog's life. CDVR seem to be best visualized at a younger age and later decline in frequency as the hypochondroplastic dog matures [7]. Hansen (1952) reported an increased frequency of histopathologically confirmed calcifications in the chondrodystrophic dogs up to the age of 7 years (77% calcified discs), with 100% of the discs in the thoracic region being histopathologically calcified at the age of 6 years. Hansen (1952) also reported a decline in histopathologically confirmed calcifications in chondrodystrophoid dogs older than 7 years old. These findings indicate that although the CDVRs will decline after the Dachshunds first years of life the histologically detectable disc calcifications may increase in number up to the age of seven. In the older Dachshunds calcifications visible on radiographs and detectable on histopathology may disappear. One could conclude that radiological examination is not the most sensitive method to detect disc calcifications and does not necessarily reflect the real extent of the degenerative process within the Dachshund discs. The results of our study support the previous findings that CDVRs are most frequently found in young adult Dachshunds (3-4 years old, mean 5.7; SD ± 3.24) and most rare in older Dachshunds (≥ 8 years old, mean 2.4; SD ± 3.42).

We found a bimodal anatomical occurrence of CDVR, with one minor peak at the transition from cervical to thoracic spine, and a larger peak at the transition from thoracic to lumbar spine (Figure 1.). This bimodal appearance has already been presented by Stigen (1996). The distribution of calcified discs on radiographs does not entirely correlate with the site of disc extrusions but seem to occur most frequently in areas of high mobility of the vertebral column (Figure 1.).

Chondroid metaplasia is a unique degenerative process of intervertebral discs seen only in hypochondroplastic dog breeds [1,2,22]. This short legged phenotype has recently been associated with the expression of a retrogene, encoding fibroblast growth factor (*Fgf4*) [23]. Subsequently, it is likely that the intervertebral disc calcification, part of this degenerative process, will show high estimates of heritability [9,10,12]. The chondroid metaplasia is inherited and all Dachshunds are affected by this degenerative process, however, not all dogs will have disc extrusions. The occurrence of IVDD is multifactorial and the fate of the degenerated hypochondroplastic disc will be affected by various factors, such as body dimensions, environmental factors including mechanics, together determining whether the disc will be subjected to clinically significant IVDD or not [21,24].

It still needs to be proven that selection of breeding dogs based on CDVR in the Dachshund has a potential to reduce the incidence of IVDD in the Dachshund population. Absence of radiological disc calcifications does not exclude degenerative changes in the disc nor does it exclude disc calcification.

Intervertebral disc calcification is undoubtedly a sign of severe disc degeneration and is a serious risk factor for the development of IVDD. Our results indicate that CDVR were more frequent in our study of Dachshunds surgically treated for disc extrusions compared to previous studies [9,11,13,14,21]. The occurrence of CDVR varies in different studies but conclusions should be made carefully as the study designs, inclusion criteria, age of examined Dachshunds, absence or presence of sedation for radiographic examination and the way radiographs were evaluated (analog or digital) might have influenced the final results [6,8,10,11,14,20]. The Dachshunds included in our study were of all sizes, hair coats and varied in age and the way they were used by the owners.

## Conclusions

In conclusion, we found that disc extrusions occur as frequently in discs that are found to have radiographic evidence of calcification as those discs that do not have signs of radiographic calcification, and that IVDD requiring surgery does occur in the absence of any calcified discs on radiographic examination. We found that calcified discs were more frequent in our Dachshund population compared to previous studies suggesting that disc calcification might be a serious risk factor for developing disc extrusion. Further studies are needed to show, conclusively, if selection of breeding dogs based on CDVR in the Dachshund will reduce the incidence of IVDD. The presence of the calcifications of intervertebral disc should be evaluated with caution, as only part of the calcifications will be detected and the real extent of the disc degeneration may be underestimated.

## Competing interests

The authors declare that they have no competing interests.

## Authors' contributions

CR drafted the manuscript, JJ and RV carried out the evaluations of the radiographs, SC designed the study, RV performed the statistical analysis and CR, JJ, RV and SC were all involved in the clinical procedures involved in the study.

All authors read and approved the final manuscript.
